# Comparative transcriptomic analysis of genes in the triterpene saponin biosynthesis pathway in leaves and roots of *Ardisia kteniophylla* A. DC., a plant used in traditional Chinese medicine

**DOI:** 10.1002/ece3.8920

**Published:** 2022-05-19

**Authors:** Yuyang Lei, AJ Harris, Aihua Wang, Liyun Zhao, Ming Luo, Ji Li, Hongfeng Chen

**Affiliations:** ^1^ Key Laboratory of Plant Resources Conservation and Sustainable Utilization South China Botanical Garden Chinese Academy of Sciences Guangzhou China; ^2^ Wuhan Guishan Mountain Scenic Management Office Wuhan China; ^3^ Key Laboratory of Environment Change and Resources Use in Beibu Gulf (Nanning Normal University) Ministry of Education Nanning China; ^4^ CAS Engineering Laboratory for Vegetation Ecosystem Restoration on Islands and Coastal Zones South China Botanical Garden Chinese Academy of Sciences Guangzhou China; ^5^ Key Laboratory of South China Agricultural Plant Molecular Analysis and Genetic Improvement South China Botanical Garden Chinese Academy of Sciences Guangzhou China; ^6^ Guangdong Provincial Key Laboratory of Applied Botany South China Botanical Garden Chinese Academy of Sciences Guangzhou China

**Keywords:** DEGs, Illumina sequencing, Primulaceae, transcription factors, triterpenoid saponins

## Abstract

*Ardisia kteniophylla* (Primulaceae) is highly valued in traditional medicine due to its production of the pharmacologically active secondary metabolites, especially triterpenoid saponins in its roots. Although *A*. *kteniophylla* is very important in traditional medicine, the genetic basis for its production of triterpenoid saponins remains largely unknown. Therefore, we sequenced transcriptomes of *A*. *kteniophylla* to identify putative genes involved in production of triterpenoid saponins in both leaves and roots, and we used the transcriptomes to compare expression levels of these genes between the two organ systems. The production of triterpenoid saponins in plants is usually induced through hormonal signaling on account of the presence of pests. Thus, we treated plants with the hormones salicylic acid (SA) and methyl jasmonate (MeJA) and used quantitative real‐time PCR (qRT‐PCR) to investigate expression levels of genes involved in triterpenoid saponin biosynthesis. In total, we obtained transcriptomes for leaf and root tissues representing 52,454 unigenes. Compared with the leaf transcriptome, we found that 6092 unigenes were upregulated in the root, especially enzymes involved in the direct synthesis of triterpenoid saponins, while 6001 genes appeared downregulated, including those involved in precursory steps in the triterpenoid saponin biosynthesis pathway. Our results from qRT‐PCR indicate that genes within the upstream parts of the triterpenoid saponin biosynthesis pathway may be upregulated under exposure to the applied hormones, but downstream genes are downregulated. This suggests possible conflicting effects of SA and MeJA in promoting the production of secondary metabolites on the one hand, and, on the other, limiting plant growth processes to devote energy to combating pests. We also performed an analysis of transcription factors (TFs) and found 997 unique transcripts belonging to 16 TF families. Our data may help to facilitate future work on triterpene saponins biosynthesis in *A*. *kteniophylla* with potential pharmacological and molecular breeding applications.

## Graphical abstract

We investigated the genetic basis for the production of triterpenoid saponins in the medicinal plant, *Ardisia kteniophylla*, using transcriptome sequences and qRT‐PCR. We found that genes involved in late stages of triterpenoid saponin biosynthesis are upregulated in roots, which are traditionally regarded as the medicinal organ of the plant, compared with leaves. We also determined that exposure to certain plant hormones, which may be involved in triggering triterpenoid saponin production, did not yield an overall upregulation of genes involved in the synthesis pathway, possibly due to energetic tradeoffs.

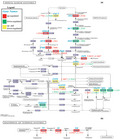

## 1 INTRODUCTION

*Ardisia kteniophylla* A. DC. is a perennial plant within the Primulaceae family (Ericales; Huang et al., 2017), and its roots are widely known to accumulate pharmacologically active products such as saponins, benzoquinones, flavanones, and sterols (Sun, Li, et al., 2017; Sun, Jiang, et al., 2017; Zhou, 2017). These products likely form the biochemical basis for the utility of *A*. *kteniophylla* in traditional Chinese medicine. In particular, the dried root of *A*. *kteniophylla* is used in southern China for the treatment of rheumatism, muscle and bone pain, and traumatic injury (Dai et al., 2017; Tang, 2007; Xiang & Feng, 2002). The medicinal value of *A*. *kteniophylla* has driven annually increasing demand for the species and has, thus, placed pressure on wild resources, which can no longer adequately supply the markets. Wild populations are also threatened by large‐scale illegal mining, which has resulted in considerable habitat loss. Therefore, unfortunately, *A*. *kteniophylla* is on the verge of extinction within China and globally due to both overharvesting and habitat destruction (Wei et al., 2015). While its conservation may be achievable based on comprehensive assessments of its genetic recourses, so far, genetic data for *A*. *kteniophylla* are lacking.

The most pharmacologically significant bioactive compounds produced by *A*. *kteniophylla* are triterpenoid saponins (Mu et al., 2010), which consist of triterpene sapogenin, sugars, uronic acid, and other organic acids. Broadly, terpenes, such as triterpenoid saponins, are found in many diverse medicinal plant species (e.g., Dong et al., 2021). Triterpene saponins have a wide range of pharmacological applications as anti‐cancer, anti‐inflammatory, anti‐allergic, and anti‐viral agents and have value for the treatment of leukemia and hypoglycemia, as well as for the prevention and treatment of cardiovascular and cerebrovascular diseases (Fleck et al., 2006; Ma et al., 2007; Ponou et al., 2008; Sparg et al., 2004; Sun et al., 2004, 2009; Vermeersch et al., 2009; Yan et al., 2006). Among saponins in *A*. *kteniophylla*, one has shown significant inhibitory effects on six different lines of tumor cells (Gu et al., 2014). Broadly, the *Ardisia* genus appears to represent a rich source of triterpene saponins (Kobayashi & De Mejía, 2005; Su et al., 2003; Zhang, 1994).

In general, the biosynthesis of saponins is well‐characterized and primarily involves three major classes of enzymes. These are *oxidosqualene cyclases* (*OSC*s), *uridin diphosphate glycosyltransferases* (*UGT*s), and *cytochrome P450 monooxygenases* (*CYP450*s; Sawai & Saito, 2011). Respectively, these classes of enzymes construct the basic triterpenoid structures, or skeletons, facilitate oxidation reactions, and catalyze the attachment of carbohydrates to a hydroxyl or similar functional groups of other molecules (i.e., glycosylation; Sawai & Saito, 2011).

In plants, saponins are part of a chemically diverse array of secondary metabolites that function in defense against microbes, diseases, and other pests (Papadopoulou et al., 1999). Plant defenses are stimulated through environmental interactions that induce signal transduction, which is often carried out by plant hormones. Among plant hormones, jasmonic acid, its methyl ester, methyl jasmonate (MeJA), and salicylic acid (SA) are endogenous and involved in signal transduction related to defense and, specifically, the production of secondary metabolites (Wang et al., 2015). When SA and MeJA are applied exogenously experimentally, they have been shown to induce upregulation of genes involved in triterpenoid saponin biosynthesis (Cao et al., 2015; Chang et al., 2016).

Unfortunately, the knowledge of genetic and hormonal regulation of biosynthesis of triterpenoid saponins within many medicinal plants, such as *A*. *kteniophylla*, is constrained by lack of genetic resources, including functionally characterized gene sequences. Characterization and new discoveries of functional genes can be expedited for medicinal plants (and other non‐model plant species) using transcriptome sequencing and de novo assembly (Liu et al., 2018; Minoche et al., 2011; Sangwan et al., 2008).

In this study, we sought to elucidate the genomic basis for the biosynthesis of triterpenoid saponins in *A*. *kteniophylla*. Specifically, our objectives were to (1) detect and characterize genes involved in biosynthesis of triterpenoid saponins using transcriptomic data resulting from RNA sequencing (RNA‐Seq), (2) compare the expression levels of genes involved in triterpenoid saponin biosynthesis in leaves and roots of *A*. *kteniophylla* to better understand the genomic mechanisms underlying disparity in accumulation of these compounds between these two organ systems, and (3) investigate differential levels of transcription of triterpenoid saponin biosynthesis under exposure to the plant hormones, SA and MeJA. Additionally, we assessed the transcription factor families represented by the transcriptomes that we sequenced from roots and leaves to infer possible mechanisms of upstream regulation of the biosynthesis of triterpene saponins in *A*. *kteniophylla*.

## 2 MATERIALS AND METHODS

### 2.1 Plant materials

We obtained seeds of *A*. *kteniophylla* from a group of plants of similar age and height from a nursery in Nanxiong, Guangdong, China. We grew the seeds on mixed soil (coconut bran: perlite: peat soil = 1:1:1) in a greenhouse at South China Botanical Garden, Guangzhou, Guangdong, China at 20–22°C, which is typical of the average annual temperature in the natural habitat of the species. Based on the preference of *A*. *kteniophylla* for dark, humid environments (Wei, 2018), we set the greenhouse conditions at 70%–80% humidity and 10%–20% ambient occlusion at all hours of the day. For optimal growth conditions, we sprayed the plants with water three times daily.

After two years of growth, we divided the seedlings of *A*. *kteniophylla* into SA and MeJA treatment groups and a control group (CK) with six plants in each group. We applied 1 mmol/L concentration of SA or MeJA to leaves of seedlings in the respective treatment groups by spraying until droplets on the leaves were dripping off, and we sprayed leaves of the CK plants with water based on protocols suggested in Werner and Schmülling (2009). All treatments occurred at 8 a.m., 12 a.m., and 6 p.m. daily for a total of seven days based on protocols successfully used for *Ardisia crenata* in a prior study (Yang, 2015).

We selected the concentrations of SA and MeJA and timings of measurements based on prior studies of the medicinal plants, especially *Panax ginseng*, *Ardisia crenata*, and *Astragalus mongholicus* (Cao et al., 2015; Chang et al., 2016; Yang, 2015). For example, the study on *Panax ginseng* (Cao et al., 2015) examined the content of medicinally important ginsenosides and found that 1 mmol/L concentration of SA was effective for their yield and quality. Similarly, a study on *Astragalus membranaceus* found that 1 mmol/L of MeJA was optimal for the species to yield saponins (Chang et al., 2016). Based on these prior findings, we performed a preliminary experiment in which we treated six seedlings of *A*. *kteniophylla* with 1 mmol/L of MeJA or SA and assessed triterpenoid saponin yield. The preliminary results supported the suitability of application of 1 mmol/L for our experiments.

Following seven days of treatments, we selected three healthy plants out of six from the CK and each treatment group (representing three biological replicates) for sampling of leaves and roots for quantitative real‐time PCR (qRT‐PCR) and spectrophotometric analysis. We performed both the qRT‐PCR and spectrophotometric analyses on the same plants. Additionally, we sampled leaves and roots for RNA extraction and subsequent Illumina sequencing from the same three CK plants. After sampling leaf and root tissues for RNA extraction, we immediately stored the samples in liquid nitrogen and kept them at −80℃ until processing.

### 2.2 RNA extraction and verification of differences in triterpene saponin concentrations in leaves and roots

We used spectrophotometry to verify that there was a difference in triterpenoid saponin concentrations between leaves and roots in *A*. *kteniophylla* sufficient to merit further downstream analyses. To prepare tissues for spectrophotometry, we first homogenized 0.1 g of each tissue sample, placed 1.0 ml of homogenate into 20 ml test tubes, and dried the samples using compressed air. We also generated a control comprising 1.0 ml of distilled water in a 20 ml tube. After the samples were dry, we performed a vanillin‐perchloric acid assay (Hiai et al., 1976) to detect the presence of triterpenoid saponins. Specifically, we added 0.2 ml of 5.0% vanillin‐glacial acetic acid solution and 1.0 ml of perchloric acid to the tissue and vortexed to mix. Thereafter, we placed the samples in a water bath at 80°C for ten minutes followed by an ice bath for five minutes before adding 8 ml of glacial acetic acid. We used a Specord210Plus spectrometer (Analytikjena) to measure absorbance of the resulting mixture at 550 nm, representing the absorption peak for oleanolic acid, which has the strongest peak among triterpenoid saponins (Hiai et al., 1976). We calculated the concentration of triterpene saponins using the following equation:

$$\text{Content of triterpenoid saponins}\;\left(\text{mg}/g\right)\;=\;\left(C*V\right)/W,$$

where *C* is the mass of triterpenoid saponins in mg/ml calculated according to the absorbance and the standard curve, *V* is the volume of the extract, and *W* is the mass of the sample.

To evaluate differences in the concentrations of triterpenoid saponins among the CK and two experimental groups, we used SPSS22.0 to perform analyses of variance (ANOVAs), and we performed *t*‐tests for assessing the differences within groups. In order to generate a standard curve for comparison with the empirical results, we used graduated amounts of oleanolic acid standard solution (1, 2, 3, 4, and 5 ml) and carried out the same color reaction. Absorbance was measured at 550 nm, and a linear regression was performed on the absorbance value A with concentration C (mg/ml).

For RNA extraction, we used 50 mg of tissue with a Huayueyang Quick RNA Isolation Kit according to the manufacturer's protocol. Following extraction, we assessed the quality, purity, and integrity of the total RNA using electrophoresis on a 1.0% (w/V) gel, a NanoPhotometer spectrophotometer, and an Agilent 2100 bioanalyzer. We required that extracted RNAs comprised a concentration of at least 200 ng/µl for downstream processing.

### 2.3 Illumina sequencing

We prepared complementary DNA (cDNA) libraries from the extracted RNAs from roots and leaves of *A*. *kteniophylla*. We generated the libraries using NEBNext^®^ Ultra™ RNA Library Prep Kit for Illumina^®^ (NEB) following the manufacturer's recommendations and attached index adaptors to each sample for downstream identification. The sequencing was performed by Novogene Bioinformatics Technology Co. Ltd., using an Illumina novaseq 6000 and paired‐end sequencing technology with a read length of 150 bp. The sequencing workflow utilized an Illumina cBot Cluster Generation System with a TruSeq PE Cluster Kit v3‐cBot‐HS. We submitted the resulting raw reads to the National Center for Biotechnology Information (NCBI) Sequence Read Archive (SRA) database (accession number PRJNA675388).

### 2.4 Transcriptome assembly and annotation

We processed the raw reads and obtained high‐quality clean sequences using Illumina Casava 1.8 (Richter & Sexton, 2009). Specifically, we removed the adapter sequences as well as reads with ambiguity (i.e., “Ns”) and/or Phred scores of ≤20 for more than 50% of bases. We performed de novo assembly of the clean, high‐quality reads using Trinity (Grabherr et al., 2011) under default settings. Trinity comprises several steps, including a finishing step, “Butterfly,” which determines the relative number of reads supporting each path along a de Bruijn graph to disentangle paralogs that were considered to represent a single gene in prior steps (Grabherr et al., 2011). In the earliest version of Trinity, this approached successfully disentangled all paralogs from 43% of multi‐gene families and distinguished two or more paralogs from 85% of multi‐gene families (Grabherr et al., 2011). Trinity is also known to outperform other assembly algorithms when paralogy is expected due to polyploidy (Chopra et al., 2014). Thus, while we cannot rule out (and fully expect) merger, or collapse, of some closely related paralogs within multi‐gene families in our assemblies, we believe that Trinity represents one of the most robust tools available for de novo assembly at present.

From the assembly, we annotated gene functions based on databases comprising NCBI non‐redundant protein (nr) and nucleotide sequences (nt), Pfam, EuKaryotic Orthologous Groups (KOG), Swiss‐Prot, Kyoto Encyclopedia of Genes and Genomes (KEGG), and Gene Ontology (GO; Table 1) primarily using Blastx, Blastn, or database‐specific tools (Conesa et al., 2005). We identified transcription factor (TF) families using iTAK (Zheng et al., 2016), which applies hmmscan (Bilmes, 2006) to compare gene sequences to a profile comprising a set of rules (Jin et al., 2014; Pérez‐Rodríguez et al., 2010), in this case, about allowed and disallowed domains in plant TFs based on annotated sequences from several databases.

**TABLE 1:** Databases, software, and parameters used in gene annotation

Database	Software and non‐default parameters
Nr	Diamond v0.8.22 e‐value = 1e−5
Nt	NCBI blast 2.2.28+ e‐value = 1e−5
Pfam	HMMER 3.0 package, hmmscan e‐value = 0.01
KOG	Diamond v0.8.22 e‐value = 1e−3
Swiss‐Prot	Diamond v0.8.22 e‐value = 1e−5
KEGG	KEGG Automatic Annotation Server e‐value = 1e−10
GO	Based on NCBI nr and Pfam results: Blast2GO v2.5 e‐value = 1e−6

### 2.5 Analysis of differentially expressed genes based on transcriptomes and qRT‐PCR

We used RSEM (Li & Dewey, 2011) to align the clean reads of each sample to the reference assembly from Trinity and determined the number of reads, or read count, aligned to each gene. We used the average of read counts from the three samples of leaves and roots to infer Foldchange.

We determined differentially expressed genes (DEGs) between the leaves and roots of three samples of *A*. *kteniophylla* representing the CK group using the DESeq package (1.10.1) in R (Love et al., 2014). DESeq infers DEGs from reads based on read count using a negative binomial distribution with mean and variance inferred via local regression. From the outcome of DESeq, we adjusted the resulting *p*‐values using the Benjamini and Hochberg's correction (Benjamini & Hochberg, 2000). We regarded genes found by DESeq with an adjusted log2Foldchange > 1 and *p*‐value < .05 as reliable DEGs between the two types of tissues. To visualize and compare foldchange, we used the R package, DEGseq R package (Wang, Feng, et al., 2010; Wang, Luan, et al., 2010), to generate a volcano plot (Li, 2012), which, broadly, is a scatter plot showing the magnitude of change (*x*‐axis) versus statistical significance (*y*‐axis).

We also compared DEGs within upstream and downstream parts of the triterpenoid saponin biosynthesis pathway between leaves and roots. For this purpose, we regarded reactions leading to the production of squalene and 2,3‐oxidosqualene from isoprene units of mevalonate (MVA) or methylerythritol phosphate (MEP) as upstream and the production of triterpene saponins from squalene and 2,3‐oxidosqualene as downstream. The production of triterpene saponins from squalene and 2,3‐oxidosqualene comprises three steps that are catalyzed by *oxidosqualene cyclases* (*OSC*s), *cytochrome P450 monooxygenases* (*CYP450*s), and *uridin diphosphate glycosyltransferases* (*UGT*s), which we anticipated finding among DEGs representing the downstream part of the pathway.

For comparing among root and leaf tissues of the CK and experimental groups, we used quantitative reverse transcriptase PCR (qRT‐PCR). We also used qRT‐PCR to verify results based on the tally of transcripts for the CK and roots of the experimental groups. For DEGs in leaves, we investigated expression levels using qRT‐PCR because the high quality of extracted RNAs from leaf tissues made this approach the most reliable.

For qRT‐PCR, we reverse‐transcribed total RNA with a GoScript™ Reverse Transcription Mix (Promega) according to the manufacturer's protocol and performed reactions using a SYBR^®^ qPCR Master Mix on a Roche LightCycler480II (Roche) with gene‐specific primer pairs. Primers were developed for this study based on the coding sequences of the genes (Table S1). As a standard, we used *Beta*‐*Actin* (*β*‐*ACTIN*; a “housekeeping” gene) and calculated its relative expression between the two types of tissue using $2^{‐\Delta \Delta C_{t}}$ (Livak & Schmittgen, 2001).

## 3 RESULTS

### 3.1 Determination of the content of triterpene saponins

**FIGURE 1:**
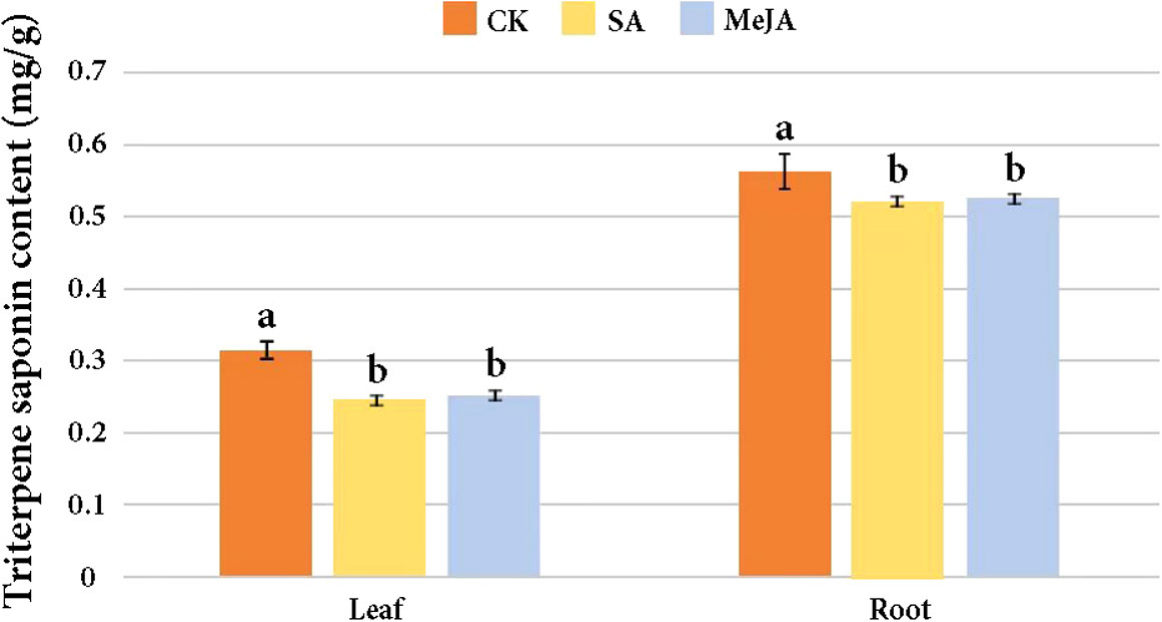
Content of triterpene saponins in different experimental and control groups. CK = Control, SA = salicylic acid, MeJA = methyl jasmonate. In each set of three bar charts, samples labeled with the same letter do not show statistically significant differences, while those bearing different letters are statistically different from one another at the α = .05 level based on a Tukey's HSD test. Error bars represent standard deviation among three biological replicates

Within both treatment groups and the CK, the content of triterpene saponins in root tissues of *A*. *kteniophylla* was much higher than in leaf tissues (Figure 1) based on spectrophotometry. In both the leaves and roots, the CK bore significantly higher concentrations of triterpene saponins than either of the treatment groups (α = 0.05). However, we observed only negligible differences in concentrations of triterpene saponins between the two treatment groups for the leaves and roots.

### 3.2 Sequencing and sequence assembly

After determining that there were differences in concentrations of triterpenoid saponins between the leaves and roots of *A*. *kteniophylla* using spectrophotometry, we performed Illumina sequencing of these tissues from CK plants representing three biological replicates from three different plants. The resulting sequences comprised an average of 22,505,663 high‐quality reads from leaves and 22,321,393 high‐quality reads from roots following filtering and 97.38% and 97.39% of sequences, respectively, had Phred quality scores of 20 (Q20) or better. The *de novo* assembly in Trinity yielded 132,319 transcripts and 52,454 unigenes (File S1) with N50 of 2719 and 2464 bp for the leaves and roots, respectively, and the mean length of transcripts and genes was 1804 and 1457 bp, respectively (Table 2).

**TABLE 2:** Summary statistics of transcriptome sequencing data for leaves and roots of *Ardisia kteniophylla*

Sample	Clean reads	Clean bases (Gb)	Error rate	Q20 percentage	Q30 percentage	GC percentage
Sequencing						
root1	22,666,075	6.8	0.03	97.6	93.14	45.81
root2	22,583,212	6.77	0.03	97.17	92.14	45.16
root3	21,714,893	6.51	0.03	97.41	92.68	45.67
leaf1	22,060,727	6.62	0.03	97.42	92.7	45
leaf2	22,079,381	6.62	0.03	97.26	92.3	44.91
leaf3	23,376,880	7.01	0.03	97.45	92.69	44.42

	Transcripts	Unigenes				
Assembly						
Total number	132,318	52,454				
Mean length (bp)	1804	1457				
N50 (bp)	2719	2464				
N90 (bp)	861	572				
Total nucleotides	238,692,243	76,400,059				

### 3.3 Functional annotation and classification

We were able to annotate all 52,454 unigenes using at least one source among the NCBI non‐redundant protein (nr) and nucleotide sequences (nt) databases, Pfam, KOG, Swiss‐Prot, KEGG, and the Gene Ontology (GO) database (Table 3). This represents a 100% success rate in annotation of unigenes. In the case of 5808 unigenes, we performed successful annotation using all databases.

**TABLE 3:** Number and percentages of 52,454 unigenes successfully annotated using each sequence database

Database	Number of unigenes	Percentage (%)
NCBI non‐redundant protein (nr)	27,400	52.23
NCBI nucleotide sequences (nt)	20,773	39.6
KEGG orthology	9743	18.57
Swiss‐Prot	22,231	42.38
Pfam	21,405	40.8
Gene Ontology (GO)	21,405	40.8
KOG	7438	14.18
Annotated by all databases	5808	11.07
Annotated by at least one database	52,454	100

**FIGURE 2:**
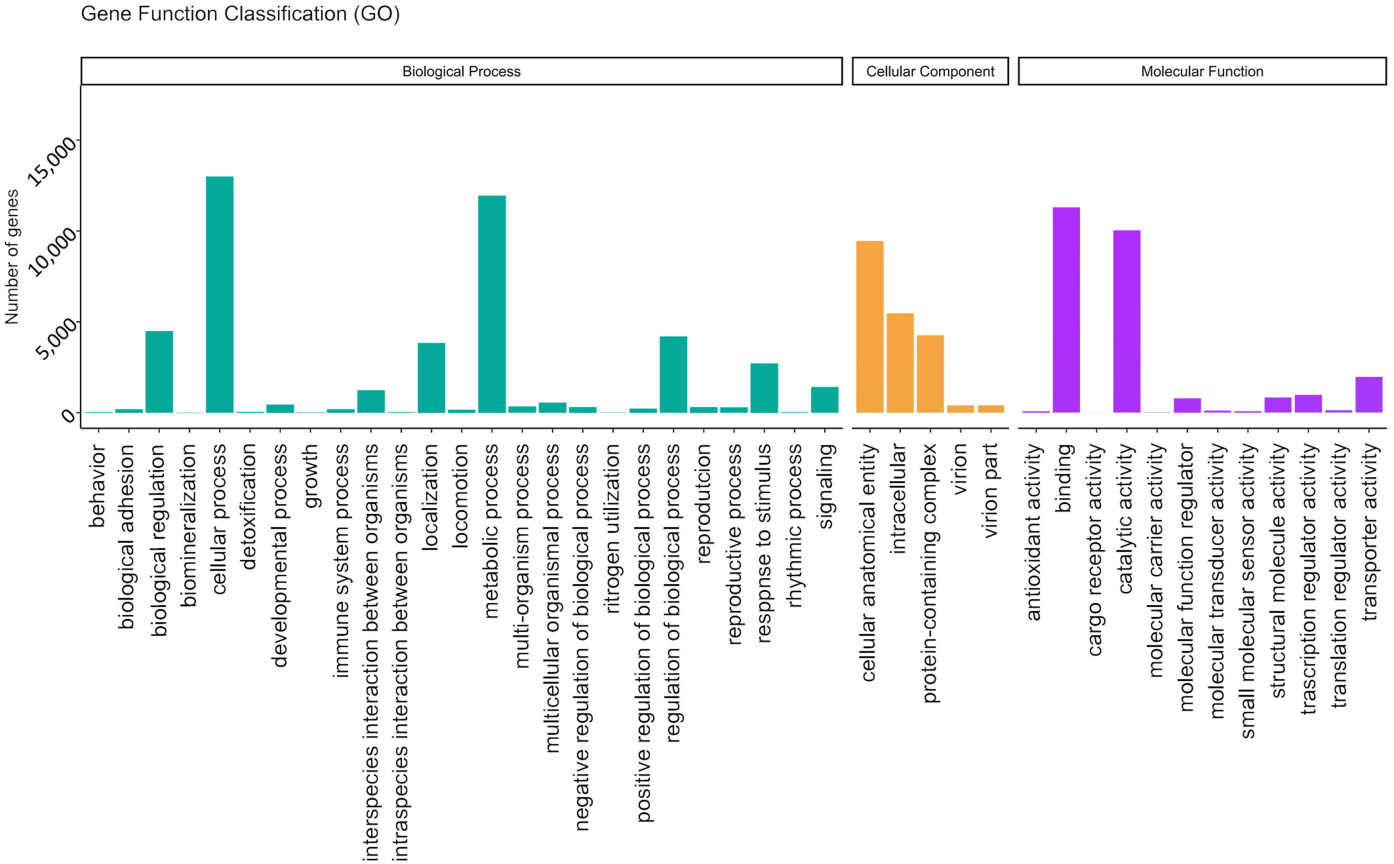
Gene Function Classification using GO. Classifications one rank down from the top‐level GO categories of Biological Process, Cellular Component, and Molecular Function are shown

In GO, the largest proportion of genes in the biological process category were annotated as “cellular process” (GO:0009987; 12,989 genes, 28.26%), within the cellular component category, the largest proportion were annotated as “cellular anatomical entity” (GO:0110165; 9457 genes, 47.20%), while “binding” (GO:0005488; 11,316 genes, 42.68%) was the most common annotation within the molecular function category (Figure 2).

**FIGURE 3:**
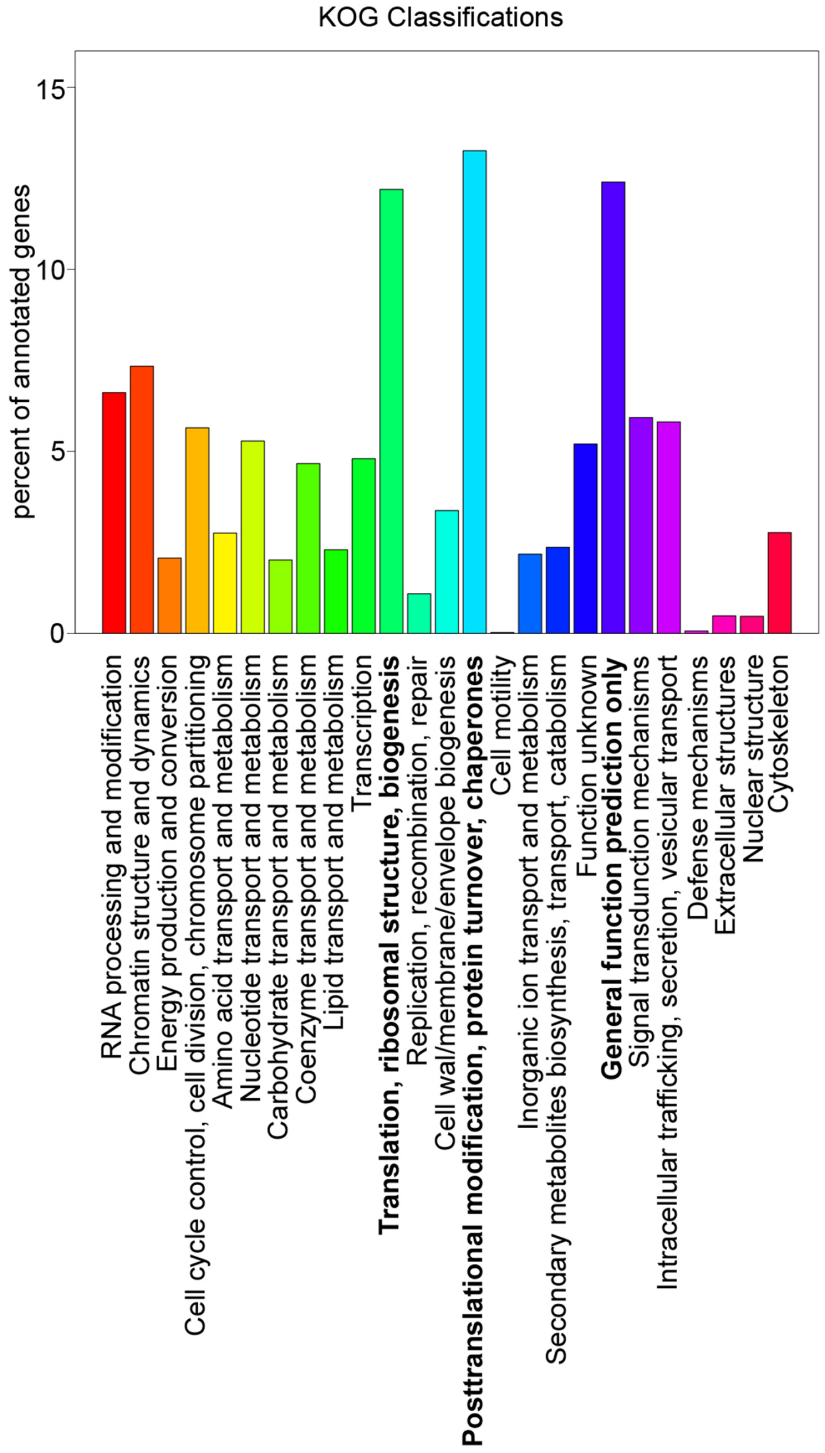
Gene Function Classification using KOG. The three classifications showing the highest enrichment are shown in bold type

In KOG, we annotated 7438 genes according to their functional categories (Figure 3). The top three categories represented by the genes were “translation, ribosomal structure and biogenesis” (907 annotated genes, 12.20%), “posttranslational modification, protein turnover, chaperones” (986 annotated genes, 13.25%), and “general function prediction only” [usually representing biochemical activity (Tatusov, 2001) 922 annotated genes, 12.40%].

**FIGURE 4:**
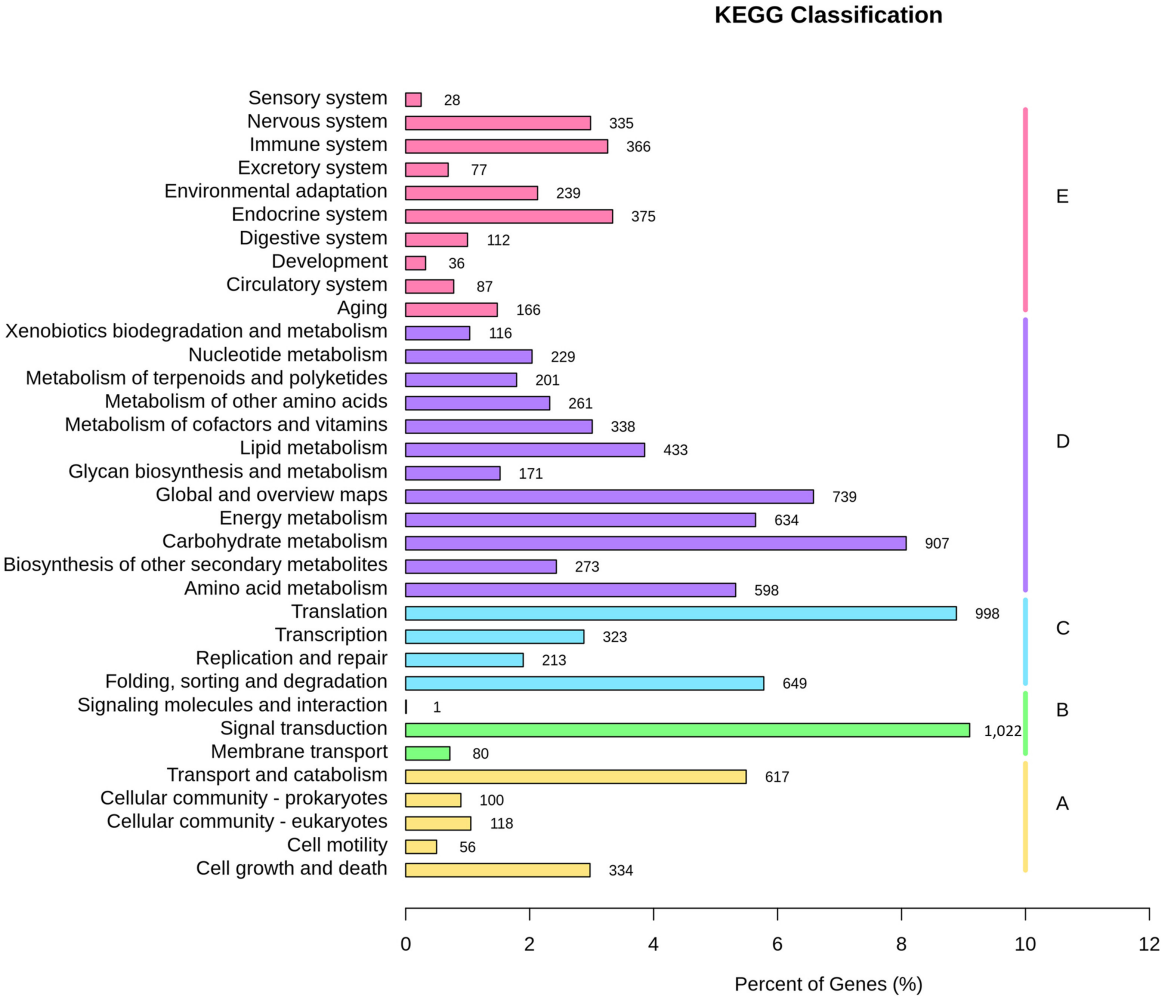
Kyoto Encyclopedia of Genes and Genomes classification of 9743 assembled unigenes. Categories of annotations to pathways shown to the right comprise cellular processes (A), environmental information processing (B), genetic information processing (C), metabolism (D), and organismal systems (E). For each pathway, the number of genes is shown to the right of the bar, which corresponds to the total percentage of genes indicated by the *x*‐axis

We used the KEGG database to map 9743 genes of *A*. *kteniophylla* to five total biochemical pathways (Figure 3). These consisted of cellular processes, environmental information processing, genetic information processing, metabolism, and organismal systems (Figure 4). For each biochemical pathway, the secondary pathways with the highest proportion of annotated genes were “transport and catabolism” (617 genes, 6.33%), “signal transduction” (1022 genes, 10.49%), “translation” (998 genes, 10.24%), “carbohydrate metabolism” (907 genes, 9.31%), and “endocrine system” (375 genes, 3.85%), respectively.

### 3.4 Differential expression analysis

**FIGURE 5:**
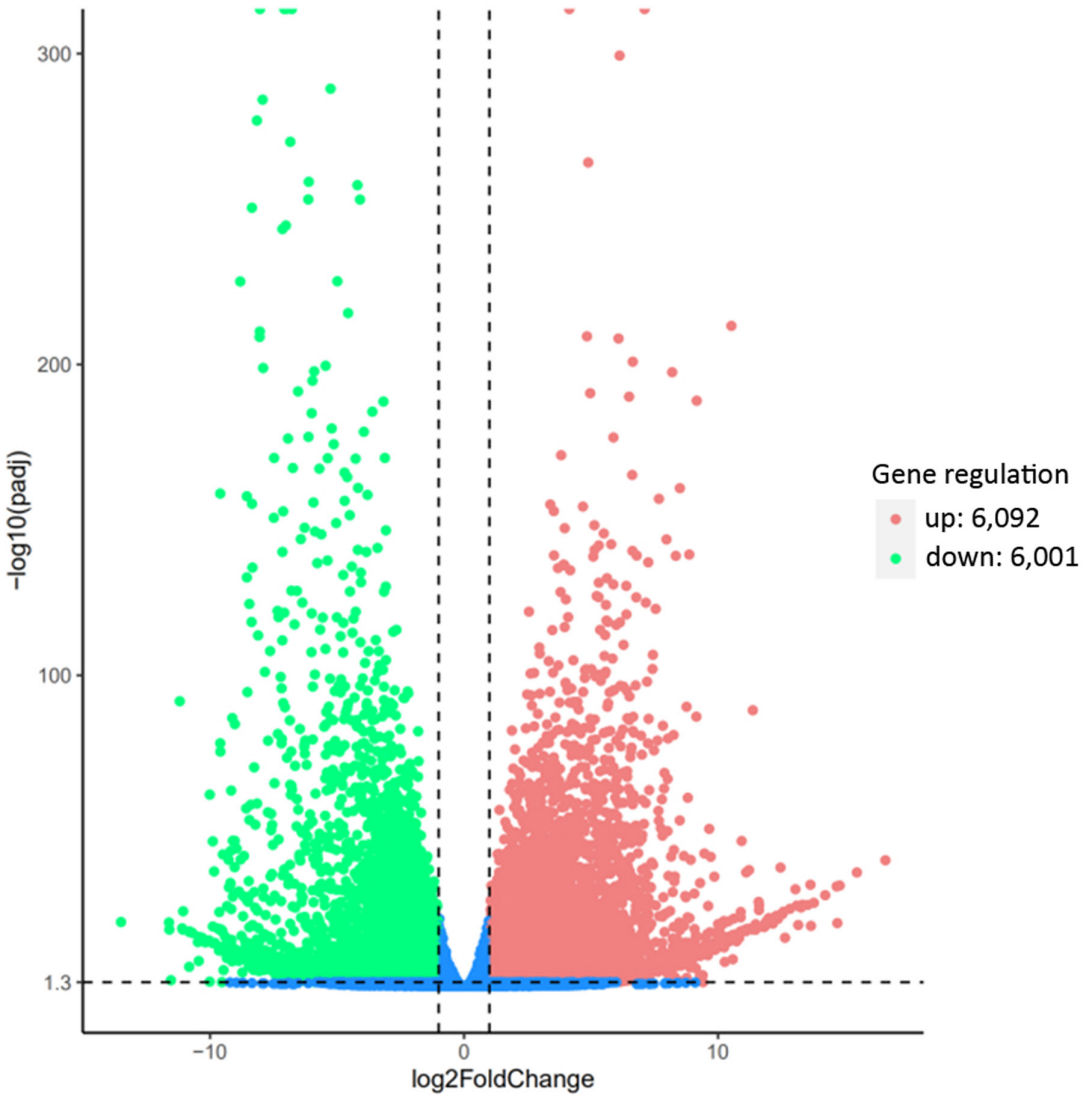
Volcano plot showing differentially expressed genes in roots of *Ardisia kteniophylla* compared with leaves. The *x*‐axis indicates the fold change in expression for different genes, and the *y*‐axis indicates the significance level of the difference in expression. The blue dashed line represents the threshold for determining that differential expression is significant

We identified DEGs between leaves and roots of *A*. *kteniophylla* in DESeq (Table S2) and detected 6092 unigenes upregulated in the roots and 6001 unigenes upregulated in leaves (Figure 5). Among DEGs, 7065 showed significant differential expression, and these represented 4307 GO terms. Among the GO terms, 2495, 606, and 1215 DEGs were involved in biological progresses, comprised cellular components, and were related to molecular functions, respectively. Within the biological progress category, the greatest portion of DEGs were mapped to “protein phosphorylation” (408 DEGs, 16.35%; GO: 0006468). For DEGs associated with the cellular component category, the majority were mapped to “tubulin complex” (76 DEGs, 12.54%; GO: 0045298), and DEGs representing molecular function were most frequently involved in “oxidoreductase activity” (852 DEGs, 70.12%; GO: 0016705; Figure 6).

**FIGURE 6:**
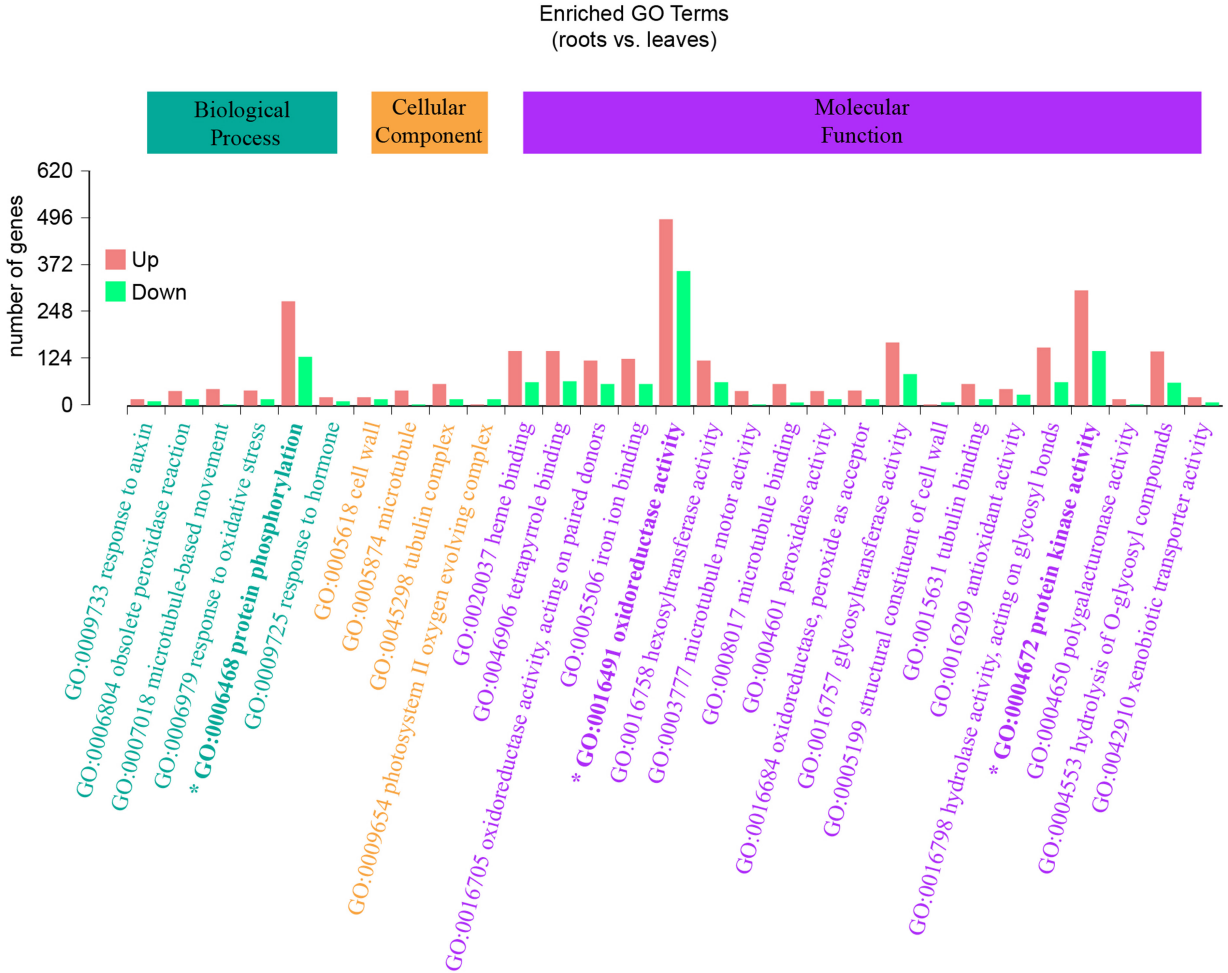
Gene Ontology annotations of DEGs in *Ardisia kteniophylla*. Terms shown on the *x*‐axis are one level below the highest‐level terms: biological process (BP), cellular component (CC), and molecular function (MF). Bold type and asterisks indicate the three terms with the largest number of DEGs overall

**FIGURE 7:**
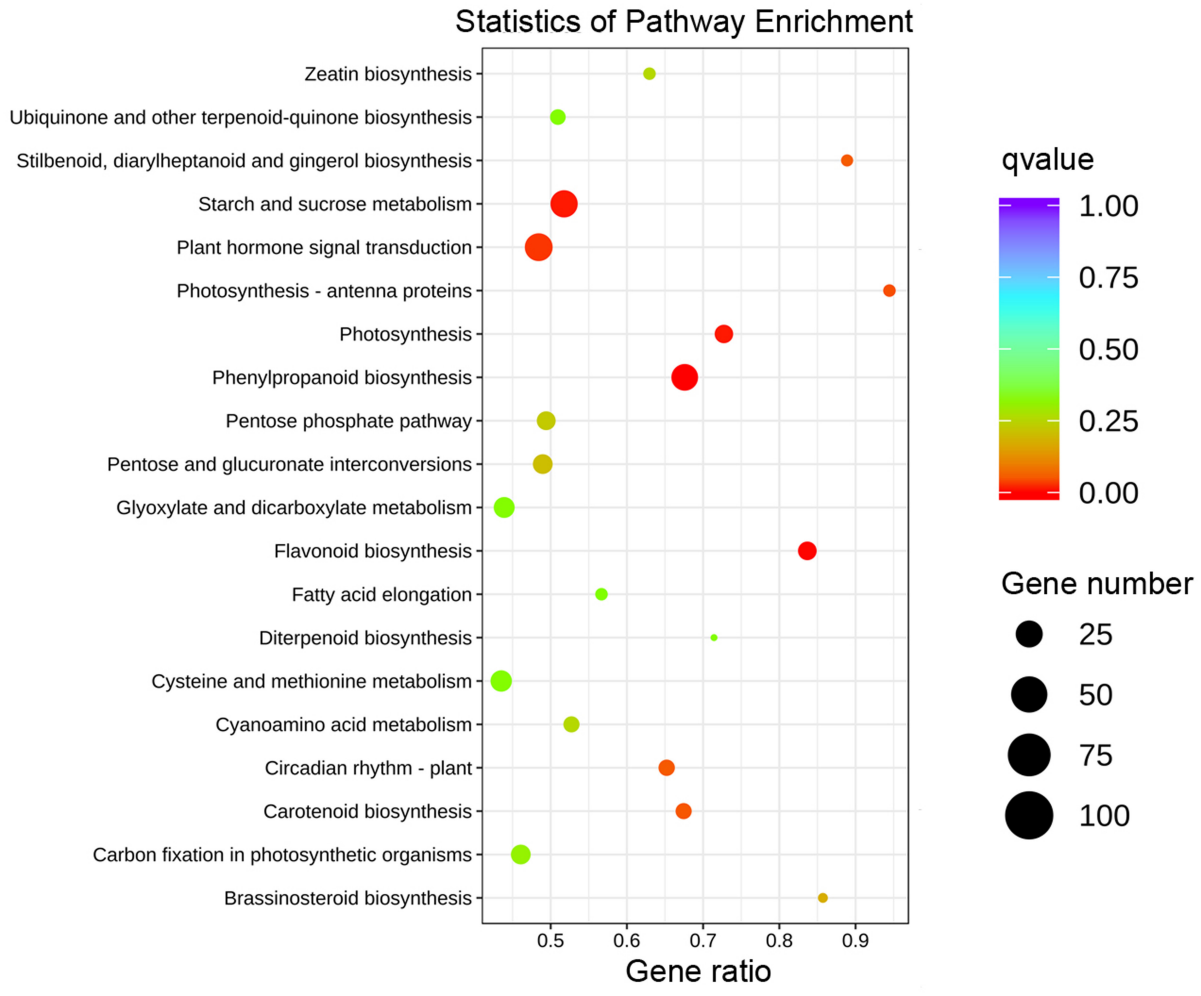
Enrichment of DEGs in KEGG pathways. The number of DEGs contained in each pathway is represented by the size of the dot

We mapped 2737 DEGs onto 117 pathways in the KEGG database. The top three pathways involving DEGs were “plant hormone signal transduction” (108, 3.95%; KO:04075), “starch and sucrose metabolism” (103, 3.76%; KO:00500), and “phenylpropanoid biosynthesis” (98, 3.58%; KO:00940). Moreover, the KEGG pathways “phenylpropanoid biosynthesis,” “flavonoid biosynthesis,” and “photosynthesis” contained DEGs showing the greatest differences between the root and leaves of *A*. *kteniophylla* based on qvalues (i.e., a type of corrected *p*‐values; Figure 7).

### 3.5 Genes in the up‐ and downstream pathways of triterpene saponin biosynthesis

**FIGURE 8:**
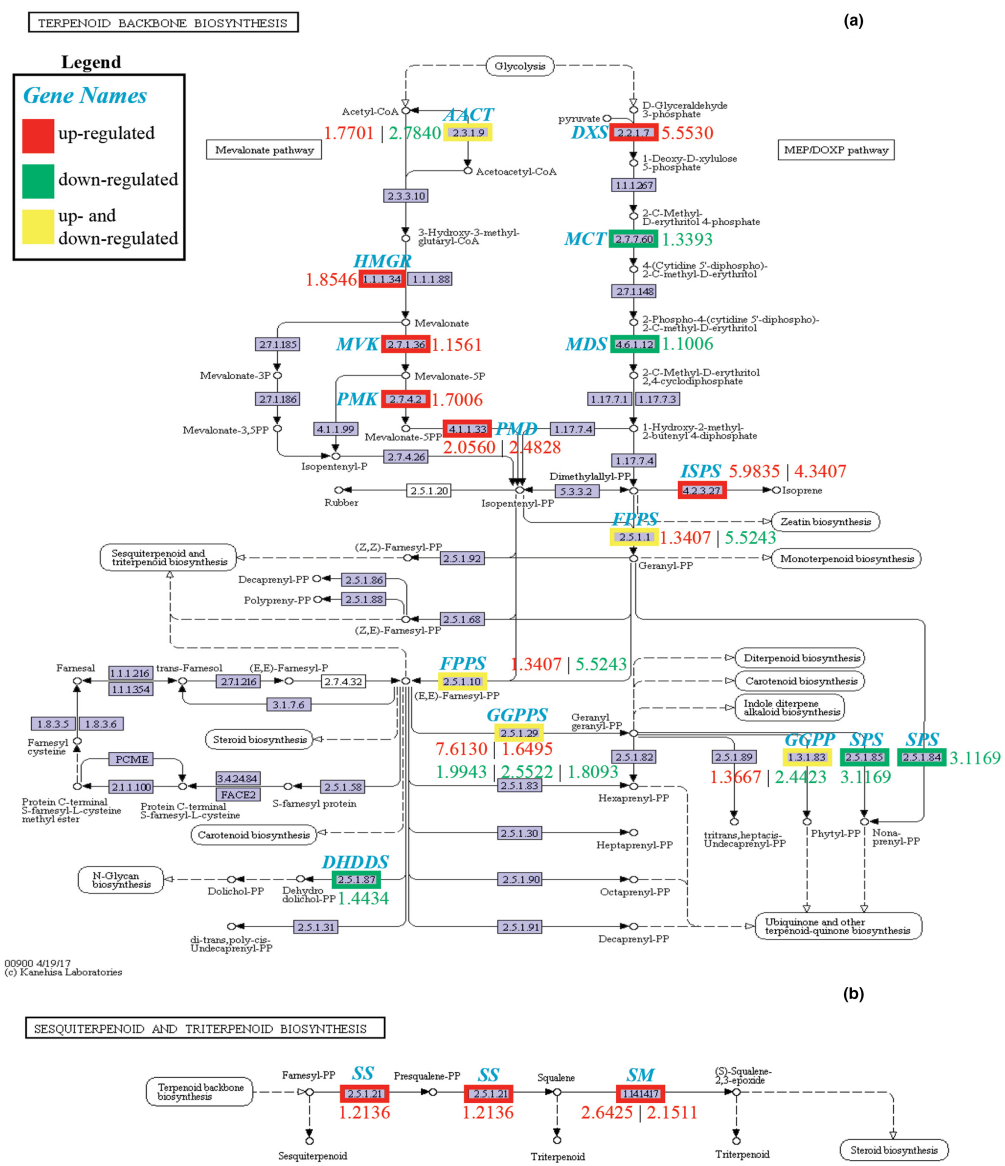
Enrichment of DEGs for the KEGG pathways. (a) terpenoid backbone biosynthesis pathway (ko00900) and (b) sesquiterpenoid and triterpenoid biosynthesis (ko00909), which are both related to triterpene saponins synthesis. Numbers below or adjacent to nodes indicate the ratio of log2 (fold change). Genes are up or downregulated in roots compared with leaves

We found that DEGs involved in the upstream MVA pathway, such as *3*‐*hydroxy*‐*3*‐*methylglutaryl*‐*CoA reductase* (*AkHMGR*), 5‐diphosphomevalonate decarboxylase (*AkPMD*), were more abundant in roots compared with the leaves. In contrast, DEGs associated with the MEP pathway, which showed greater abundance of genes in leaves, included *2*‐*C*‐*methyl*‐*D*‐*erythritol*‐*2*,*4*‐*cyclodip*‐*hosphate synthase* (*AkMDS*) and *4*‐*hydroxy*‐*3*‐*methylbut*‐*2*‐*enyldiphosphate synthase* (*AkHDS*) (Table S3). Based on log2 (fold change), we found that genes involved in the final steps of upstream biosynthesis processes, such as *squalene synthase* (*AkSS*) and *squalene monooxygenase* (*AkSM*), were more highly expressed in roots, while leaves showed greater expression of genes involved in biosynthesis of earlier precursory compounds (Figure 8).

From among DEGs in the downstream part of the triterpene saponin biosynthesis pathway, we identified two *OSCs*, 144 *CYP450*s, and 31 *UGT*s, of which 0, 100, and 22 satisfied the criterion of log2 fold change ≥1, respectively. Overall, more DEGs in the downstream pathway were more highly expressed in roots compared with leaves (Table S4). None of the predicted *CYP450s* or *UTG*s were annotated to the KEGG pathways for triterpenoid biosynthesis (Figure 8), and this may be because the functions of these genes are still broadly unknown and are not presently included in the focal pathways by KEGG.

### 3.6 Quantitative reverse transcriptase PCR

**FIGURE 9:**
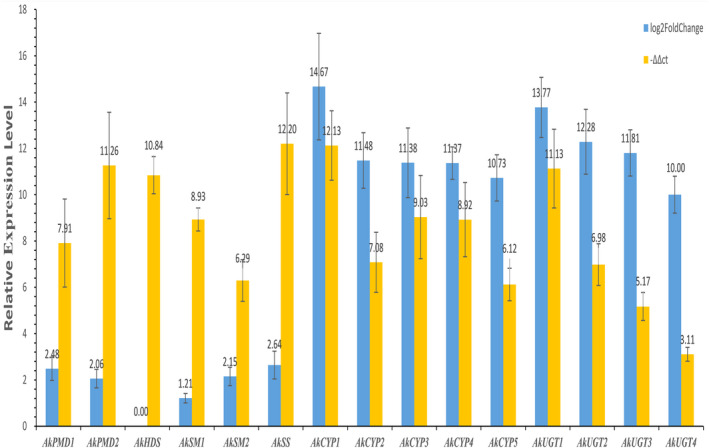
Relative expression levels of DEGs selected for qRT‐PCR and transcriptome in roots relative to leaves of *Ardisia kteniophylla*. Comparison of log2Foldchange in the transcriptome data with the −ΔΔC*_t_* in the qRT‐PCR experiment. Error bars represent the standard deviation among three biological and three technical replicates

Based on the DEGs inferred from transcriptome sequences, we selected the following genes for qRT‐PCR: two *AkPMD* unigenes, one *AkHDS* unigene, one *AkSS* unigene, two *AkSM* unigenes, five *AkCYP450s* unigenes, and four *AkUGTs* unigenes. We found that all genes analyzed using qRT‐PCR were upregulated in the roots of the CK plants (Figure 9) as is generally consistent with the DEGs inferred from the transcriptomes (Table S1).

**FIGURE 10:**
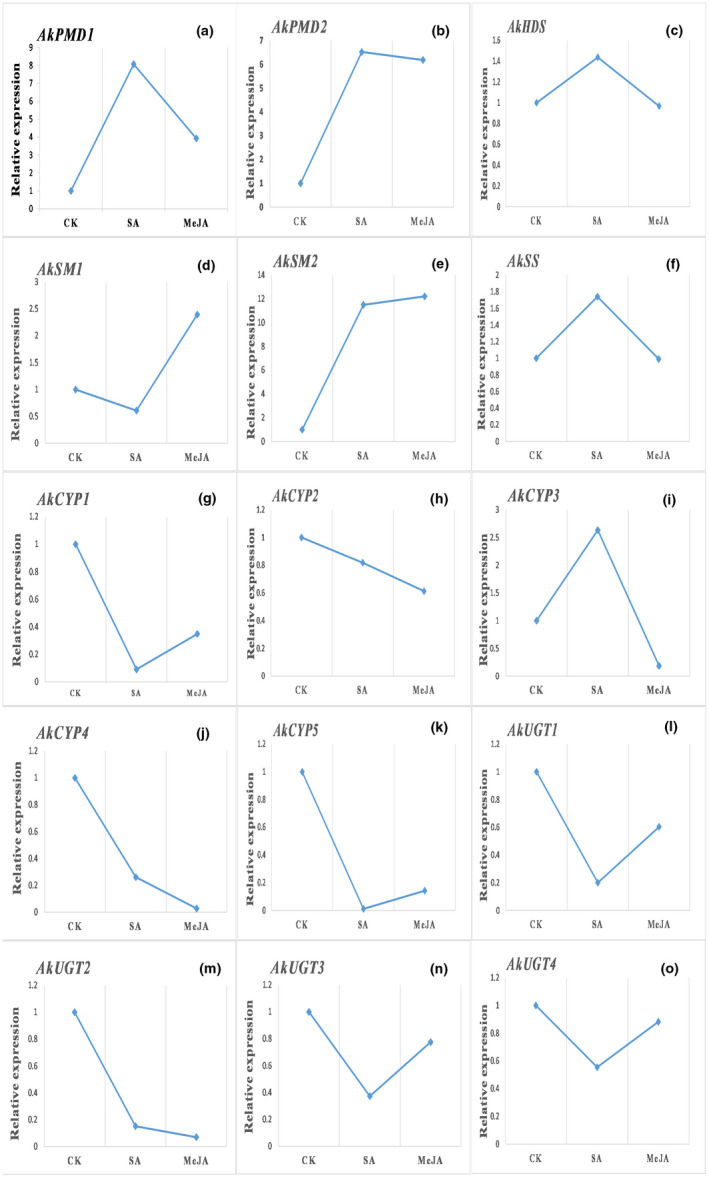
Relative expression levels of DEGs selected for qRT‐PCR in hormone treatment group and control group. For comparisons, we set the relative abundance of each gene in control group to 1.0. Each point represents the average of three biological replicates

Compared with the CK plants, genes operating upstream within the triterpene saponin biosynthesis pathway (*AkPMDs*, *AkHDS*, *AkSMs*, and *AkSS*) were upregulated in both the SA and MeJA treatment groups (Figure 10). In contrast, all genes involved in the downstream pathway (*AkCYP450s* and *AkUGTs*) showed downregulation in the treatment groups compared with the CK except *AkCYP3*, which was upregulated under SA treatment (Figure 10i). Overall, regulation of genes in the up‐ and downstream pathways showed greater up‐ or downregulation in response to SA than to MeJA.

### 3.7 Prediction of transcription factors

In total, we annotated 997 transcription factors in *A*. *kteniophylla*, and, of these, 590 were upregulated in the root and 401 were upregulated in leaves (Table 4 and Table S5). In particular, members of the *Myeloblastosis* (*MYB*) and *Apetala 2*/*Ethylene Response Factor* (*AP2*/*ERF*) TF families were the most highly upregulated in roots with 95 and 76 members, respectively. Notably, *MYB* was also the most highly upregulated in leaves with 65 upregulated members. Overall, we found that the 1865 potential TFs accounted for 3.56% of the *A*. *kteniophylla* unigene library.

**TABLE 4:** Differentially expressed transcription factor families in *Ardisia kteniophylla*

Transcription factor family	Upregulated in root	Upregulated in leaf	Total detected
*AP2/ERF*	76	37	114
*NAC*	54	37	91
*C2H2*	50	29	80
*MYB*	95	65	161
*bHLH*	51	27	79
*WRKY*	36	33	69
*bZIP*	29	23	52
*C3H*	22	34	56
*GRAS*	26	17	43
*SNF2*	24	17	41
*B3*	21	20	41
*GARP*	22	17	39
*LOB*	18	9	29
*SET*	24	13	37
*Trihelix*	21	8	29
*PHD*	21	15	36

## 4 DISCUSSION

### 4.1 Triterpenoid saponin biosynthesis genes of *A*.* kteniophylla* and their expression levels in leaves and roots

In *A*. *kteniophylla*, we detected a larger number of unigenes over 1 kb in length compared with its congener, *Ardisia crenata* (Yang, 2015); 23,568 versus 14,659, respectively. The difference in our results might be due to the availability of more data within public databases in contrast to six years ago when the prior study was published and to improvements in transcriptome sequencing capabilities (Stark et al., 2019). The long lengths of unigenes generated in this study suggest an overall high‐quality transcriptome (Wang, Feng, et al., 2010; Wang et al., 2012; Wang, Luan, et al., 2010) that is likely responsible for our 100% success rate at annotating all 52,454 unigenes that we recovered.

We detected 2737 DEGs between leaves and roots of *A*. *kteniophylla* and mapped these to117 pathways in the KEGG database (Figure 7). Of the 2737 DEGs, 108 (3.95%) were mapped to the “plant hormone signal transduction” pathway (KO:04075), and this was the largest number of genes mapped to any one pathway. Notably, genes participating in signal transduction may induce the expression of key enzymes that are involved in the production of secondary metabolites (Arimura et al., 2000), such as triterpene saponins (Yendo et al., 2014). Several specific signal transduction pathways influencing the production of triterpene saponins have been studied in medicinal species of *Panax*, and signaling mechanisms in those species include calcium, ethylene, and nitric oxide, and reactive oxygen species (Rahimi et al., 2015). However, more research is needed to directly connect the signal transduction enzymes that we detected in the roots and leaves of *A*. *kteniophylla* to levels of accumulation of triterpene saponins.

Our results show that the two pathways for generation of 2,3‐oxidosqualene from isoprene units occur differentially in the leaves and roots based on DEGs. Within the roots, the MVA pathway is more active in the generation of 2,3‐oxidosqualene from isoprene units, while, in leaves, the MEP pathway is more active. These findings were consistent with previous studies, which have shown that genes representing the MVA pathway are most highly expressed in the radicle, hypocotyl, roots, flowers, and seeds, whereas genes of the MEP pathway are more active in leaves (Vranová et al., 2013). This is likely because the MEP pathway is confined to plastids (Lichtenthaler, 1999; Rodríguez‐Concepción et al., 2004), which are usually most abundant in the leaves as chloroplasts. Moreover, while the MEP pathway may be technically operational within plastids that have not been exposed to light, that is, etioplasts, precursory components for initiating the MEP pathway appear to be lacking in dark environments, thus limiting the utility of this pathway in roots (Nagata et al., 2002).

Differentially expressed genes in the downstream part of the triterpene saponin biosynthesis were, overall, more highly expressed in roots compared with leaves (Table S4). This is consistent with higher concentrations of triterpene saponins within the roots of *A*. *kteniophylla* based on prior studies using biochemical assays (Wei, 2017) and our own results (Figure 1). Moreover, prior studies using gene expression analysis have often shown higher levels of gene expression in the roots of medicinal plants compared with leaves (e.g., *Medicago truncatula*, Fabaceae; Huhman et al., 2005), even in cases where the leaf is the organ preferred for pharmacology (e.g., *Hedera helix*, Araliaceae; Sun, Li, et al., 2017; Sun, Jiang, et al., 2017).

Notably, roots may often be the final site of biosynthesis and storage of triterpene saponins (Sawai and Saito, 2011), while earlier, upstream stages of synthesis occur in leaves (and other tissues not sampled in this study, such as stems, rhizomes, and flowers) as shown in the medicinal plants, *Platycodon grandiflorum* (Campanulaceae; Ma et al., 2016) and *Panax japonicus* (Araliaceae; Rai et al., 2016). In the medicinal herb, *Panax ginseng*, *CYP450*s are more highly expressed in rhizomes, while *UGT*s are more highly expressed in roots, suggesting these organs may be, respectively, the intermediate and final sites of triterpene saponin biosynthesis (Han et al., 2012; Rai et al., 2016). Overall, plant roots have often been shown to accumulate higher concentrations of secondary metabolites, such as triterpenoid saponins, and this may be due to the complex nature of the soil environment, where roots are forced into close contact with potentially harmful microbes and pests, thus necessitating strong defenses (Su et al., 2005).

### 4.2 Expression of triterpenoid saponin biosynthesis genes of *A*.* kteniophylla* under exposure to SA and MeJA plant hormones

We found that exogenous application of the signal transduction hormones, SA and MeJA, yielded an overall decrease in the content of triterpene saponins compared with the CK (Figure 1). Research in other plant groups has shown that exogenous application of MeJA can increase the production of triterpenoid saponins compared with control plants, such as in *Centella asiatica* (Apiaceae; Mangas et al., 2006). Similar results were found in *Nigella sativa* (Ranunculaceae) under exogenous application of SA (Elyasi et al., 2016). However, both hormones are known to limit plant growth, potentially resulting in negative interactions between growth‐related pathways and production of secondary compounds (Ellis & Turner, 2001; Rudell et al., 2002). The degree to which plants are able to both increase secondary compound production while plant growth is stunted under SA or MeJA is likely taxon‐specific. For example, in *Ginkgo biloba*, the production of terpene trilactones was increased under SA application even while genes involved in photosynthesis (and, thus growth) were downregulated (Ye et al., 2020), and this is in contrast to our results. In taxa where there is stunting of plant growth, this may represent a trade‐off where survival in the face of pests is given precedence.

In the case of *A*. *kteniophylla*, application of SA and MeJA appeared to upregulate genes involved in the early stages of biosynthesis of triterpene saponins compared with the CK, but the critical downstream gene families, *P450* and *UGT*, were downregulated. There is a known antagonistic relationship between *P450* and the effects of MeJA on the production of secondary metabolites involved in defense in *Arabidopsis* (Lee et al., 2011), which can be further explored in future studies within *A*. *kteniophylla*. Overall, exogenous application of SA or MeJA to leaves of *A*. *kteniophylla* does not improve the production of the pharmacologically valuable triterpenoid saponins in the species, suggesting that more work is needed to understand the complexities of the biosynthesis pathway for these compounds.

### 4.3 Transcription factors families that may regulate triterpenoid saponin biosynthesis in *A*.* kteniophylla*

Our analyses revealed that TFs previously reported present in *A*. *kteniophylla*, *WRKY*, *AP2*/*ERF*, *NAC*, *MYB*, and *basic helix–loop–helix* (*bHLH*; De Geyter et al., 2012; Yang et al., 2012), were always more active in roots. For example, 76% of genes in the *AP2*/*ERF* family that we identified were more highly expressed in roots and similarly with 54% of *NAC* genes. Moreover, the high overall prevalence of TFs in the genome of *A*. *kteniophylla* that we can infer from our transcriptomes (i.e., 3.56%) is roughly consistent with *Arabidopsis*, in which 1500 TFs comprise ca. 5% of the genome (Riechmann et al., 2000). A slight difference here may be due to a larger number of TFs detected within the whole genome of *Arabidopsis* compared with our utilization of transcriptomes.

Members of the *AP2*/*ERF* TF family have been shown to regulate secondary metabolic pathways in several medicinal plants. For example, in *Artemisia annua* (Asteraceae), two *AP2*/*ERF* TFs, *AaERF1* and *AaERF2*, are responsive to jasmonic acid (JA) and positively regulate genes encoding amorpha‐4,11‐diene synthase and *CYP71AV1*, enzymes directly involved in biosynthesis of the secondary metabolite, artemisinin (Yu et al., 2012), which is widely used medicinally. Similarly, the *AP2*/*ERF* TFs, *ORCA2* and *ORCA3*, in *Catharanthus roseus* (Apocynaceae) regulate the metabolism of pharmacologically valuable terpenoid indole alkaloids by activating the expression of the strictosidine synthase gene (Menke et al., 1999; Van Der Fits and Memelink, 2001). In *A*. *kteniophylla*, the large percentage of detected *AP2*/*ERF* TFs that were more highly expressed in roots may indicate a critical role of this TF family in the downstream stages of triterpenoid saponin biosynthesis for this species. Thus, *AP2*/*ERF* genes may represent critical targets for molecular breeding of this species with improved concentrations of medicinally active compounds.

## 5 CONCLUSIONS

In summary, in this study, we performed transcriptomic analysis to investigate the genetic basis for accumulation of triterpene saponins in roots and leaves of *A*. *kteniophylla*. We generated a large dataset of unigenes and successfully annotated these for structure and function based on several public databases. Our comparative analyses revealed that many genes involved in triterpene saponin biosynthesis have higher expression in roots than leaves, and roots are the primary organ used pharmacologically in traditional Chinese medicine. Our transcriptomic data provide a valuable genetic resource for this plant and may facilitate molecular breeding to meet demand for the medicinally bioactive triterpene saponins of the species.

## AUTHOR CONTRIBUTIONS

**Yuyang Lei:** Conceptualization (equal); Formal analysis (lead); Investigation (lead); Writing – original draft (lead); Writing – review & editing (equal). **AJ Harris:** Project administration (supporting); Writing – review & editing (equal). **Aihua Wang:** Formal analysis (supporting). **Liyun Zhao:** Formal analysis (supporting); Investigation (supporting); Writing – original draft (supporting). **Ming Luo:** Conceptualization (supporting). **Ji Li:** Investigation (supporting). **Hongfeng Chen:** Conceptualization (equal); Project administration (lead); Writing – review & editing (equal).

## CONFLICT OF INTEREST

The authors declare that the research was conducted in the absence of any commercial or financial relationships that could be construed as a potential conflict of interest.

## Supporting information

Table S1

Table S2

Table S3

Table S4

Table S5

File S1

## Data Availability

Illumina short reads are available from NCBI as PRJNA675388. All other raw data are included as supporting information.
